# Evidence for methionine-sulfoxide-reductase gene transfer from Alphaproteobacteria to the transcriptionally active (macro)nucleus of the ciliate, *Euplotes raikovi*

**DOI:** 10.1186/s12866-014-0288-1

**Published:** 2014-11-25

**Authors:** Nicoleta Dobri, Annalisa Candelori, Francesca Ricci, Pierangelo Luporini, Adriana Vallesi

**Affiliations:** Laboratory of Eukaryotic Microbiology and Animal Biology, School of Biosciences and Veterinary Medicine, University of Camerino, Camerino, MC, 62032 Italy

**Keywords:** Horizontal gene transfer, Protozoa, Alphaproteobacteria, Gene structure, Methionine sulfoxide reductases

## Abstract

**Background:**

Deleterious phenomena of protein oxidation affect every aerobic organism and methionine residues are their elective targets. The reduction of methionine sulfoxides back to methionines is catalyzed by methionine-sulfoxide reductases (Msrs), enzymes which are particularly active in microorganisms because of their unique nature of individual cells directly exposed to environmental oxidation.

**Results:**

From the transcriptionally active somatic genome of a common free-living marine protist ciliate, *Euplotes raikovi*, we cloned multiple gene isoforms encoding Msr of type A (MsrA) committed to repair methionine-S-sulfoxides. One of these isoforms, in addition to including a MsrA-specific nucleotide sequence, included also a sequence specific for a Msr of type B (MsrB) committed to repair methionine-R-sulfoxides. Analyzed for its structural relationships with MsrA and MsrB coding sequences of other organisms, the coding region of this gene (named *msrAB*) showed much more significant relationships with Msr gene coding sequences of Rhodobacterales and Rhizobiales (Alphaproteobacteria), than of other eukaryotic organisms.

**Conclusions:**

Based on the fact that the *msrAB* gene is delimited by *Euplotes*-specific regulatory 5′ and 3′ regions and telomeric C_4_A_4_/G_4_T_4_ repeats, it was concluded that *E. raikovi* inherited the coding region of this gene through a phenomenon of horizontal gene transfer from species of Alphaproteobacteria with which it coexists in nature and on which it likely feeds.

**Electronic supplementary material:**

The online version of this article (doi:10.1186/s12866-014-0288-1) contains supplementary material, which is available to authorized users.

## Background

Methionine residues of polypeptide chains are common targets of oxidation phenomena which alter conformation, sub-cellular localization, and aggregation state of proteins causing detrimental effects on vital cell functions and activities [1,2]. Aerobic organisms thus urgently need to repair their methionine-oxidized proteins and their minimal gene sets systematically include Msr genes [3,4].

Increasing concentrations of water-borne signaling proteins (pheromones), which *Euplotes raikovi* uses to promote its vegetative (mitotic) growth and the sexual phenomenon of conjugation [5], were observed to undergo oxidation in cause-effect relationships with cell ageing [6]. This oxidation hits the methionine residues that are more exposed on the surface of the pheromone molecular structure and was shown to cause remarkable modifications of protein activity [6], as is the case in other cell systems [2]. To shed light on the molecular mechanism evolved by *E. raikovi* to repair its methionine-oxidized pheromones, attention was focused on the Msr genes that are transcribed in the cell somatic nucleus (macronucleus) characterized by an eccentric sub-chromosomic organization in which individual, gene-size DNA molecules are replicated in thousands of copies fully autonomous for both replication and transcription [7].

Differently from the MsrB gene showing a single form, the gene specifying MsrA was found to be present in the *E. raikovi* macronucleus in multiple isoforms [8]. One isoform, designated as *msrAB* gene, is described here for its unique nucleotide sequence containing information for the synthesis of MsrA and MsrB proteins characterized by unequivocal structural relationships with MsrA and MsrB of Alphaproteobacteria.

## Results and discussion

The *msrAB* gene cloning involved two PCR steps. A 231-bp MsrA-specific DNA fragment was first generated through amplification of total DNA preparations run with a combination of degenerate oligonucleotides (labeled #1 and 2 in Additional file 1: Table S1) specific to amino acid sequence stretches conserved in MsrA proteins of various organisms. In a second step, two nested PCR amplifications were run using primers (from #3 to #6 in Additional file 1: Table S1) specific to this DNA fragment in combination with a primer (#7 in Additional file 1: Table S1) specific to the C_4_A_4_/G_4_T_4_ repeats that are distinctive of the telomeric ends of every *Euplotes* macronuclear gene-size molecule [7]. Among four structurally distinct gene isoforms that were obtained, we reconstructed the full-length sequence of the longest isoform (1595 bp) by overlapping the individual sequences. The reconstructed sequence was then confirmed by sequencing the amplification product of a PCR run with primers (#8 and 9 in Additional file 1: Table S1) specific to regions located close to its telomeric ends.

**Figure 1:**
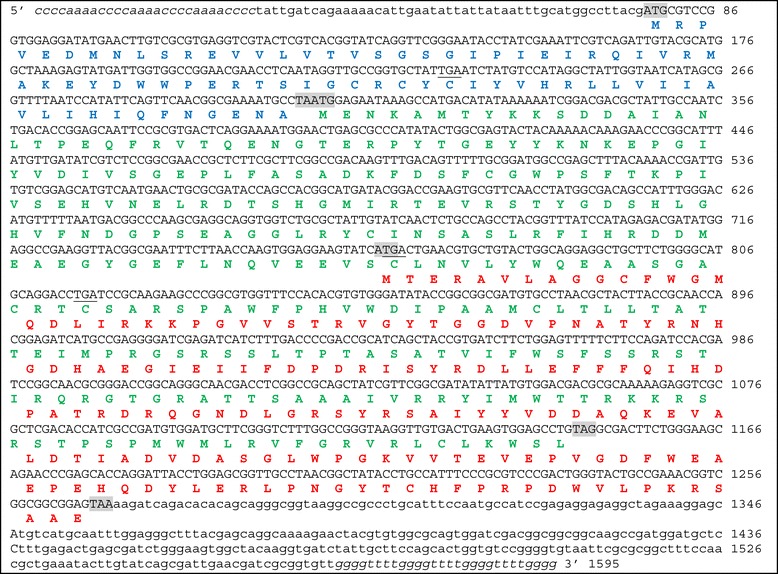
**Structure of the** ***Euplotes raikovi msrAB*** **gene.** Nucleotide sequence: telomeric repetitions, italics; the 5′ and 3′ non-coding regions, lower case letters; coding regions, capital letters; in-frame TGA codons, underlined; ATG, TAA and TAG start and stop codons, shadowed. Deduced amino acid sequence: blue, green and red letters distinguish the putative proteins encoded by the three ORFs.

Instead of including a single open reading frame (ORF) like the other three gene isoforms (obtained incomplete at their 3′ regions), the 1595-bp isoform exceptionally included three potential ORFs (Figure 1).

**Figure 2:**
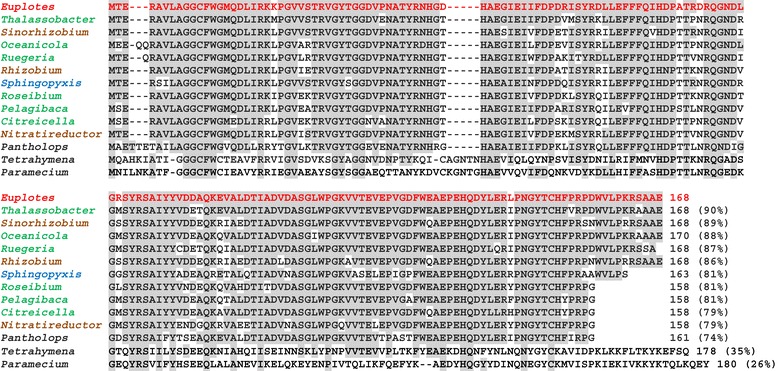
**Sequence alignment of the** ***Euplotes raikovi*** **MsrA protein (red) encoded by ORF-1 of the** ***msrAB*** **gene with MsrAs of other organisms.** MsrAs included in the alignment represent the best hits obtained from prokaryotic and eukaryotic BLASTp searches. Gaps were inserted to maximize alignment, and identical residues are highlighted in gray. Numbers in brackets indicate the percentage of sequence identity of each amino acid sequence with *E. raikovi* MsrA. Aligned sequences have the following GenBank ID: *Thalassobacter arenae*, WP_021102447; *Sinorhizobium fredii*, YP_006401320; *Oceanicola sp*., WP_010137233; *Ruegeria lacuscaerulensis*, WP_005979692; *Rhizobium sp*., WP_018236324; *Sphingopyxis sp.*, WP_003045039; *Roseibium sp*., WP_009759924; *Pelagibaca bermudensis*, WP_007796742; *Citreicella sp*., WP_008887323; *Nitratireductor aquibiodomus*, WP_007008964; *Pantholops hodgsonii*, XP_005978873; *Paramecium tetraurelia*, XP_001431627; *Tetrahymena thermophila,* XP_001020577. Rhodobacterales, green; Rhizobiales, brown; Sphingomonadales, blue; eukaryotic organisms, black.

The first ORF (ORF-1), spanning from ATG at position 763 to TAA at position 1269, matched the ORF of the three other gene sequences (Additional file 2: Figure S1). It encodes a 168-amino acid MsrA protein showing a much closer structural identity (79^_^90%) to MsrAs of Rhodobacterales, such as *Thalassobacter* (re-classified as *Litoreibacter* [9]) and *Oceanicola*, and Rhizobiales such as *Sinorhizobium*, than to any eukaryotic MsrA those of ciliates such as *Tetrahymena* and *Paramecium* included (Figure 2).

**Figure 3:**
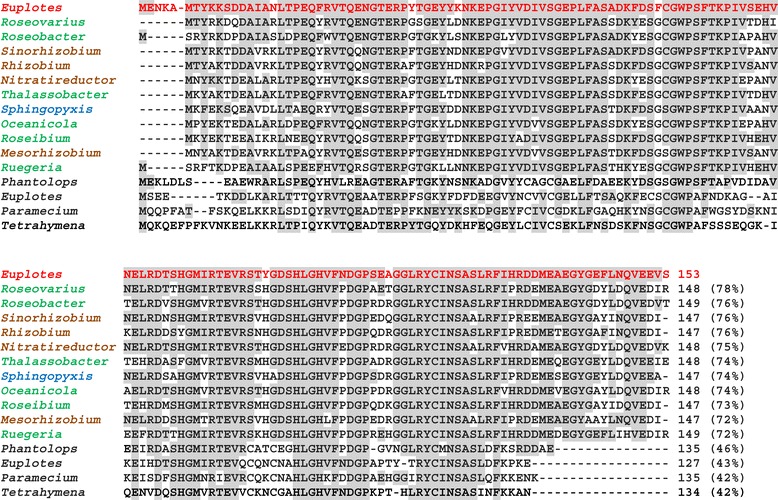
**Sequence alignment of the 153-amino acid N-terminal region of the** ***Euplotes raikovi*** **MsrB protein (red) encoded by ORF-2 of the** ***msrAB*** **gene with MsrBs of other organisms.** The MsrBs included in the alignment represent the best hits obtained from prokaryotic and eukaryotic BLASTp searches. Gaps were inserted to maximize alignment, and identical residues are highlighted in gray. Numbers in brackets indicate the percentage of sequence identity of each amino acid sequence with *E. raikovi* MsrB. Aligned sequences have the following GenBank ID: *Roseovarius nubinhibens*, WP_009814088; *Roseobacter sp*., WP_007811995; *Sinorhizobium meliloti*, WP_018098563; *Rhizobium sp*., WP_018236325; *Nitratireductor aquibiodomus*, WP_007008963; *Thalassobacter arenae*, WP_021102446; *Sphingopyxis sp*., WP_003044951; *Oceanicola granulosus*, WP_007254905; *Roseibium sp*., WP_009759925; *Mesorhizobium alhagi,* WP_008840482; *Ruegeria conchae*, WP_010442903; *Pantholops hodgsonii* mitochondrial-like, XP_005955290; *Euplotes raikovi*, AFZ61875; *Paramecium tetraurelia*, XP_001426263; *Tetrahymena thermophila*, XP_001019714. Rhodobacterales, green; Rhizobiales, brown; Sphingomonadales, blue; eukaryotic organisms, black.

The second ORF (ORF-2), spanning from ATG at position 305 to TAG at position 1150 and partially overlapping with ORF-1, includes (at the beginning of the overlapping region) an in-frame TGA codon which, however, is most likely not committed to stop translation. At least in principle, it should code for cysteine, or selenocysteine, so as TGA usually does in *Euplotes* [10]. The 152-amino acid N-terminal region of the 281-amino acid sequence encoded by this ORF shows significant relationships not with other MsrA proteins, but with bacterial MsrBs lacking Cys-Xxx-Xxx-Cys Zn-ion binding motifs [3,4]. Its alignment is much closer (72^_^78% of structural identity) to MsrBs of Rhodobacterales such as *Roseovarius*, *Roseobacter*, *Thalassobacter* and *Oceanicola*, and Rhizobiales such as *Sinorhizobium* and *Rhizobium*, than to any eukaryotic MsrB including the MsrB of *E. raikovi* itself (Figure 3).

The third ORF (ORF-3), spanning from ATG at position 78 to TAA at position 305 and containing another in-frame TGA_,_ encodes a 75-amino acid protein not related to Msr proteins. Its 40-amino acid N-terminal segment is 55^_^60% identical to the C-terminal sequence of the LysR-type transcription regulator of *Rhizobium*, *Sinorhizobium*, and *Sphingopyxis*. In addition to being strongly conserved among Rhizobiales and Sphingomonadales [11,12], this regulatory protein is known to be determined by genes carried by DNA regions destined to be transferred from one to another bacterial genome [11].

To obtain evidence that the *msrAB* gene is a functional and effectively expressed gene, cDNA preparations were obtained from cells previously induced to increase their anti-oxidative enzyme synthesis by a mild oxidative stress (generated by a 30-min suspension with 300-μM H_2_O_2_ concentration), and subjected to PCR amplification with primer combinations specific to each ORF (Additional file 3: Figure S2). Two MsrA-specific 368-bp and 660-bp products were obtained, indicating that ORF-1 is either the only one to be expressed, or is expressed to a much higher extent than the other two ORFs.

**Figure 4:**
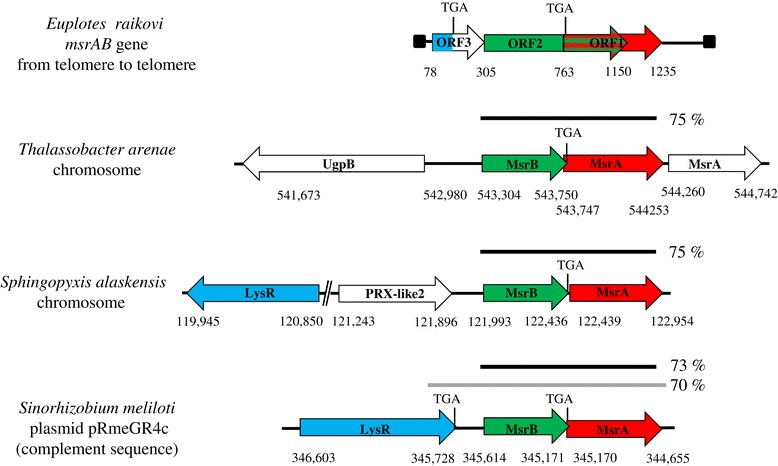
**Comparative structural analysis of the** ***Euplotes raikovi msrAB*** **gene with the MsrA and MsrB coding genes of** ***Thalassobacter arenae*** **,** ***Sinorhizobium meliloti*** **, and** ***Sphingopyxis alaskensis.*** ORFs are represented by arrows pointing to the direction of transcription and extending between the indicated nucleotide positions. Red, green and blue colors highlight MsrA, MsrB and LysR-transcription regulator ORF, respectively. Gray and black bars indicate regions of bacterial genes with 70 and 73-75% of nucleotide sequence identity with *msrAB* gene, respectively (see also Additional file 4: Figures S3-S5). Inter-ORF bars indicate non-coding regions and their relative extensions, while the filled boxes in the *msrAB* gene indicate the telomeric ends. *T. arenae*, *S. meliloti* and *S. alaskensis* sequence GenBank accession numbers are GCA_000442275.1, CP003936.1 and CP000356.1, respectively.

The bacterial origin of the three ORFs of the *E. raikovi* macronuclear *msrAB* gene is well explained by a comparative analysis with the organization of the MsrA, MsrB, and transcription-regulator gene sequences in *Thalassobacter arenae*, *Sinorhizobium meliloti* and *Sphingopyxis alaskensis* genomes [13-18]. In all these Alphaproteobacteria, the MsrB and MsrA coding genes lie adjacent to one another and the TGA stop codon of the MsrB coding region partially overlaps (*T. arenae*), or is separated by none (*S. meliloti*), or only two nucleotides (*S. alaskensis*) from the ATG start codon of the MsrA coding region (Figure 4 and Additional file 4: Figures S3-S5). In addition, in *T. arenae* and *S. alaskensis* the transcription-activator gene is located apart from the Msr coding genes [13,18]. In *S. meliloti*, instead, the distance is only 114-bp from the ATG of the MsrB coding region [14] and the MsrA/MsrB/transcription-activator gene cluster does not lie in the chromosome, but in one of the two symbiotic mega-plasmids (or chromids) [14-17].

## Conclusions

Genome analysis from a large variety of pro- and eukaryotes indicates that gene transfer among the three domains of life is a recurrent phenomenon in biological evolution. It also suggests that eukaryotic genomes preferentially retain those prokaryotic genes which encode enzymes capable of conferring adaptive and evolutionary advantages [19-21]. The finding that *E. raikovi* uses Msrs from Alphaproteobacteria to repair methionine-oxidized proteins supports these concepts, and implies that ciliates in general expand their genetic resources from the acquisition of bacterial gene sequences.

The pervasive tendency of *Euplotes* species to host endosymbiotic bacteria in their cytoplasm [22], and the fact that Rhizobiales include numerous symbiotic species [23] would suggest that the origin of the *msrAB* coding sequence lies in some *Sinorhizobium* species living as endosymbionts in *E. raikovi*. However, present-day stable cytoplasmic hosts of *E. raikovi* appear to be Gammaproteobacteria, in primis *Francisella endociliophora* [24,25], which have Msr genes with sequences markedly different from those of the *E. raikovi msrAB* coding sequences (personal communication from Dr. Andreas Sjödin, CBRN Defence and Security Department, Swedish Defence Research Agency, Umeå).

An alternative hypothesis accounting for the origin of the *msrAB* gene is suggested by the Doolittle’s aphorism “you are what you eat” [26]. It considers that the origin of the *msrAB* gene resides in some Rhodobacterales or Rhizobiales species that are usually ingested as food by *E. raikovi*. Molecular investigations and cultivation-based studies have consistently revealed that both Rhodobacterales of the so-called “marine alpha group” and Rhizobiales of the genus *Rhizobium* are cosmopolitan and dominant members of microbial communities in marine sediments [27-31]. Furthermore, they contribute to the Mediterranean subsurface microbial community of which *E. raikovi* is a common member [32].

## Methods

### Cell cultures

*Euplotes raikovi* cultures used in this study derive from the wild-type strain #13 deposited at the ATCC Center (catalog, #PRA-327), and collected (June 1979) from a sandy coastal site (Porto Recanati, 43° 26′N, 13° 43′E) of the Adriatic cost of Italy [32]. They were fed on green algae, *Dunaliella tertiolecta*, grown in pasteurized natural seawater enriched with Walne medium.

### DNA purification and amplification

Total DNA preparations were obtained, according to a published procedure [33], from cultures deprived of food for 3–4 days and concentrated by centrifugation (2500 x g, for 5 min). Degenerate primers were designed with the “CODEHOP” (Consensus-Degenerate Hybrid Oligonucleotide Primers) method [34] on the basis of the following two MsrA conserved sequence stretches: Leu-Ala-Gly-Gly-Cys-Phe-Trp and His-Asp-Pro-Thr-Thr-Leu-Asn-Arg-Gln-Gly. All the PCR amplifications were run in an Eppendorf Mastercycler (Eppendorf, AG, Hamburg, Germany), using 0.5-μg DNA aliquots as template in 50 μl-reaction mixtures containing 0.25 μM of each primer, 0.3 mM dNTP, 1x buffer, and 1U of Perfect-Taq DNA Polymerase (Eppendorf). After an initial DNA denaturation step at 95°C for 4 min, 35 cycles of 95°C for 30 sec, 58°C for 40 sec, and 72°C for 1 min were run. A final incubation step, at 72°C for 7 min, was added to the last cycle. Gel-purified PCR products were ligated into pGEM-T Easy Vector (Promega, WI) and transformed into TOPO 10 cells (Invitrogen, Life Technologies Corporation, Carlsbad, CA, USA). Colonies were selected for PCR amplification to screen the presence of inserts using standard “M13” primers and the products were sequenced at the “BMR Genomics” Center of the University of Padua.

### RNA extraction and cDNA synthesis

RNA was extracted from cells incubated with H_2_O_2_ (300 μM), for 30 min, harvested by centrifugation, and lysed in Trizol reagent (Ambion, Life Technologies Corporation, Carlsbad, CA, USA). It was then purified with the PureLink RNA mini kit (Ambion) following the procedure described by the manufacturer, and digested with RNAse-free DNAse I to remove contaminating DNA. Single-stranded cDNA was synthesized following the 3′ RACE protocol of the FirstChoice RLM-RACE kit (Ambion) and 50 ng-aliquots were next used in PCR analysis.

### Sequence analysis and accession number

BLAST analysis (www.ncbi.nlm.nih.gov/BLAST/) and ClustalW (www.genome.jp/tools/clustalw/) were used to search for the nearest relative sequences and perform multiple sequence alignments, respectively. The *msrAB* sequence has been deposited to GenBank under the accession number KM197136.

## Additional files

Additional file 1: Table S1. PCR primer numbers, denominations, and sequences. Additional file 2: Figure S1. Nucleotide **(a)** and amino acid **(b)** sequence alignments of the three *E. raikovi* MsrA gene isoforms obtained (incomplete at the 3′ regions) together with the *mrsAB* gene. Additional file 3: Figure S2.
**(a)** Schematic representation of the *msrAB* gene showing the relative positions of the three ORF’s and PCR primers used for assessing the expression of each ORF. **(b)** Agarose-gel separation of PCR products obtained from cDNA amplifications run with the indicated primer combinations. Additional file 4: Figure S3. Nucleotide sequence alignment between *E. raikovi msrAB* gene (from nucleotide 305 to nucleotide 1,268) and *Thalassobacter arenae* chromosome (from nucleotide 543,304 to nucleotide 544,253). **Figure S4.** Nucleotide sequence alignment between *E. raikovi msrAB* gene (telomeric C_4_A_4_-G_4_T_4_ repeats excluded) and *Sinorhizobium meliloti* plasmid pRmeGR4c (from nucleotide 344,364 to nucleotide 345,900, complement sequence). **Figure S5.** Nucleotide sequence alignment between *E. raikovi msrAB* gene (from nucleotide 305 to nucleotide 1,280) and *Sphingopyxix alaskensis* chromosome (from nucleotide 121,993 to nucleotide 122,954).

## References

[1] Friguet B (2006). Oxidized protein degradation and repair in ageing and oxidative stress. FEBS Lett.

[2] Stadtman ER, Moskovitz J, Levine RL (2003). Oxidation of methionine residues of proteins: biological consequences. Antioxi Redox Sign.

[3] Delaye L, Becerra A, Orgel L, Lazcano A (2007). Molecular evolution of peptide methionine sulfoxide reductases (MsrA and MsrB): on the early development of a mechanism that protects against oxidative damage. J Mol Evol.

[4] Zhang XH, Weissbach H (2008). Origin and evolution of the protein-repairing enzymes methionine sulphoxide reductases. Biol Rev.

[5] Vallesi A, Giuli G, Bradshaw RA, Luporini P (1995). Autocrine mitogenic activity of pheromones produced by the protozoan ciliate *Euplotes raikovi*. Nature.

[6] Alimenti C, Vallesi A, Luporini P, Buonanno F, Ortenzi C (2012). Cell aging-induced methionine oxidation causes an autocrine to paracrine shift of the pheromone activity in the protozoan ciliate, *Euplotes raikovi*. Exp Cell Res.

[7] Jahn CL, Klobutcher LA (2002). Genome remodeling in ciliated protozoa. Annu Rev Microbiol.

[8] Dobri N, Oumarou EE, Alimenti C, Ortenzi C, Luporini P, Vallesi A (2013). Methionine sulfoxide reduction in ciliates: characterization of the ready-to-use methionine sulfoxide-R-reductase genes in *Euplotes*. Gene.

[9] Kim YO, Park S, Nam BH, Kang SJ, Hur YB, Kim DG, Oh TK, Yoon JH (2012). Description of *Litoreibacter meonggei* sp. nov., isolated from the sea squirt *Halocynthia roretzi*, reclassification of *Thalassobacter arenae* as *Litoreibacter arenae* comb. nov. and emended description of the genus *Litoreibacter* Romanenko et al. 2011. Int J Syst Evol Microbiol.

[10] Turanov AA, Lobanov AV, Formenko DE, Morrison HG, Sogin ML, Klobutcher LA, Hatfield DL, Gladyshev VN (2009). Genetic code supports targeted insertion of two amino acids by one codon. Science.

[11] Pérez-Rueda E, Collado-Vides J (2001). Common history at the origin of the position-function correlation in transcriptional regulators in archaea and bacteria. J Mol Evol.

[12] Maddocks SE, Oyston PCF (2008). Structure and function of the LysR-type transcriptional regulator (LTTR) family proteins. Microbiology.

[13] Riedel T, Fiebig A, Petersen J, Gronow S, Kyrpides NC, Göker M, Klenk HP (2013). Genome sequence of the *Litoreibacter arenae* type strain (DSM 19593^T^), a member of the *Roseobacter* clade isolated from sea sand. Stand Genomic Sci.

[14] Galardini M, Mengoni A, Brilli M, Pini F, Fioravanti A, Lucas S, Lapidus A, Cheng JF, Goodwin L, Pitluk S, Land M, Hauser L, Woyke T, Mikhailova N, Ivanova N, Daligault H, Bruce D, Detter C, Tapia R, Han C, Teshima H, Mocali S, Bazzicalupo M, Biondi EG (2011). Exploring the symbiotic pangenome of the nitrogen-fixing bacterium *Sinorhizobium meliloti*. BMC Genomics.

[15] Barnett MJ, Fisher RF, Jones T, Komp C, Abola AP, Barloy-Hubler F, Bowser L, Capela D, Galibert F, Gouzy J, Gurjal M, Hong A, Huizar L, Hyman RW, Kahn D, Kahn ML, Kalman S, Keating DH, Palm C, Peck MC, Surzycki R, Wells DH, Yeh KC, Davis RW, Federspiel NA, Long SR (2001). Nucleotide sequence and predicted functions of the entire *Sinorhizobium meliloti* pSymA megaplasmid. Proc Natl Acad Sci U S A.

[16] Galardini M, Pini F, Bazzicalupo M, Biondi EG, Mengoni A (2013). Replicon-dependent bacterial genome evolution: the case of *Sinorhizobium meliloti*. Genome Biol Evol.

[17] Martínez-Abarca F, Martínez-Rodríguez L, López-Contreras JA, Jiménez-Zurdo JI, Toro N (2013). Complete genome sequence of the alfalfa symbiont *Sinorhizobium/Ensifer meliloti* strain GR4. Genome Announc.

[18] Lauro FM, McDougald D, Thomas T, Williams TJ, Egan S, Rice S, DeMaere MZ, Ting L, Ertan H, Johnson J, Ferriera S, Lapidus A, Anderson I, Kyrpides N, Munk AC, Detter C, Hang CS, Brown MV, Robb FT, Kjelleberg S, Cavicchioli R (2009). The genomic basis of trophic strategy in marine bacteria. Proc Natl Acad Sci U S A.

[19] Rocha EPC (2013). With a little help from Prokaryotes. Science.

[20] Keeling PJ, Palmer JD (2008). Horizontal gene transfer in eukaryotic evolution. Nat Rev Genet.

[21] Keeling PJ (2009). Functional and ecological impacts of horizontal gene transfer in eukaryotes. Curr Opin Genet Dev.

[22] Görtz HD (2001). Intracellular bacteria in ciliates. Int Microbiol.

[23] Engelhardt T, Sahlberg M, Cypionka H, Engelen B (2013). Biogeography of *Rhizobium radiobacter* and distribution of associated temperate phages in deep subseafloor sediments. ISME J.

[24] Schrallhammer M, Schweikert M, Vallesi A, Verni F, Petroni G (2011). Detection of a novel subspecies of *Francisella noatunensis* as endosymbiont of the ciliate *Euplotes raikovi*. Microb Ecol.

[25] Sjödin A, Öhrman C, Bäckman S, Lärkeryd A, Granberg M, Lundmark E, Karlsson E, Nilsson E, Vallesi A, Tellgren-Roth C, Stenberg P, Thelaus J: **Complete genome sequence of Francisella endociliophora strain FSC1006, isolated from a laboratory culture of the marine ciliate*****Euplotes raikovi.*** *Genome Announc* 2014, **2:**e01227–14. 10.1128/genomeA.01227-14 PMC4246165 25428973

[26] Doolittle WF (1998). You are what you eat: a gene transfer ratchet could account for bacterial genes in eukaryotic nuclear genomes. Trends Genet.

[27] Gonzàlez JM, Moran MA (1997). Numerical dominance of a group of marine bacteria in the α-subclass of the class Proteobacteria in coastal seawater. Appl Environ Microbiol.

[28] Macián MC, Arahal DR, Garay E, Ludwig W, Schleifer KH, Pujalte MJ (2005). *Thalassobacter stenotrophicus* gen. nov., sp. nov., a novel marine *α*-proteobacterium isolated from Mediterranean sea water. Int J Syst Evol Microbiol.

[29] Dang H, Li T, Chen M, Huang G (2008). Cross-ocean distribution of *Rhodobacterales* bacteria as primary surface colonizers in temperate coastal marine waters. Appl Environ Microbiol.

[30] D’Hondt S, Jørgensen BB, Miller DJ, Batzke A, Blake R, Cragg BA, Cypionka H, Dickens GR, Ferdelman T, Hinrichs KU, Holm NG, Mitterer R, Spivack A, Wang G, Bekins B, Engelen B, Ford K, Gettemy G, Rutherford SD, Sass H, Skilbeck CG, Aiello IW, Guèrin G, House CH, Inagaki F, Meister P, Naehr T, Niitsuma S, Parkes RJ, Schippers A (2004). Distributions of microbial activities in deep subseafloor sediments. Science.

[31] Süss J, Schubert K, Sass H, Cypionka H, Overmann J, Engelen B (2006). Widespread distribution and high abundance of *Rhizobium radiobacter* within Mediterranean subsurface sediments. Environ Microbiol.

[32] Miceli C, Luporini P, Bracchi P (1981). Morphological description, breeding system, and nuclear changes during conjugation of *Euplotes raikovi* Agamaliev from Mediterranean Sea. Acta Protozool.

[33] Vallesi A, Di Pretoro B, Ballarini P, Apone F, Luporini P (2010). A novel protein kinase from the ciliate *Euplotes raikovi* with close structural identity to the mammalian intestinal and male-germ cell kinases: characterization and functional implications in the autocrine pheromone signaling loop. Protist.

[34] Rose TM, Henikoff JG, Henikoff S (2003). CODEHOP (COnsensus-DEgenerate Hybrid Oligonucleotide Primer) PCR primer design. Nucleic Acids Res.

