# Supplementary material for: Evidence for methionine-sulfoxide-reductase gene transfer from Alphaproteobacteria to the transcriptionally active (macro)nucleus of the ciliate, Euplotes raikovi
Source: BMC Microbiol. 2014 Nov 25;14:288. doi: 10.1186/s12866-014-0288-1 (PMC4247871; doi:10.1186/s12866-014-0288-1)
Supplement: Additional file 1: Table S1. — PCR primer numbers, denominations, and sequences. [file 12866_2014_288_MOESM1_ESM.pdf]

Additional file 1

Table S1 PCR primer numbers, denominations and sequences

Numbers	Denominations	Sequences (5'-3')
#1	msrA-FW1[a]	TTCGCAGGAGGNTGHTTYTGG
#2	msrA-RV1[a]	CCTTGTCTATTAAGAGTDGTNGGRTCRTG
#3	msrA-FW2	CATGCAGGACCTGATCCGCAAGAA
#4	msrA-RV2	CAGAAGATCACGGTAGCTGATGC
#5	msrA-FW3	GCATCAGCTACCGTGATCTTCTG
#6	msrA-RV3	TTCTTGCGGATCAGGTCCTGCATG
#7	TEL	CCCCAAAACCCCAAAACCCC
#8	MsrAB-5'FW	CCCCTATTGATCAGAAAAACATTG
#9	MsrAB-3'RV	GATACAAGTATTTCAGCGTTGGAAAG
#10	MsrAB FWa	GTGGAGGATATGAACTTGTC
#11	MsrAB RVa	TTAGGCATTTTCGCCGTTGAA
#12	MsrAB FWb	ATCGGACGACGCTATTGCCAATC
#13	MsrAB RVb	TAGGCTGGCAGAGTTGATACAAT
#14	MsrAB FWc	TGCGTTCAACCTATGGCGACAG
#15	MsrAB RVc	GAAGATCACGGTAGCTGATG
#16	MsrAB RVd	CGTTTCGGCAGTACCCAGTC

[a]Degenerate oligonucleotides. Y and R: alternatives between C and T, and A and G, respectively. H and D: alternatives among A, C and T, and A, G and T, respectively. N: any nucleotide.
