# Supplementary material for: Evidence for methionine-sulfoxide-reductase gene transfer from Alphaproteobacteria to the transcriptionally active (macro)nucleus of the ciliate, Euplotes raikovi
Source: BMC Microbiol. 2014 Nov 25;14:288. doi: 10.1186/s12866-014-0288-1 (PMC4247871; doi:10.1186/s12866-014-0288-1)
Supplement: Additional file 2: Figure S1. — Nucleotide (a) and amino acid (b) sequence alignments of the three E. raikovi MsrA gene isoforms obtained (incomplete at the 3′ regions) together with the mrsAB gene. [file 12866_2014_288_MOESM2_ESM.pdf]

Additional file 2

Figure S1 Nucleotide (**a**) and amino acid (**b**) sequence alignments of the three MsrA gene isoforms obtained (incomplete at the 3' regions) together with the *msrAB* gene. Gaps were inserted to maximize alignments and sequence identities to the *msrAB* gene and to the MsrA protein encoded by *msrAB* gene are highlighted in gray. Nucleotide sequences: telomeric repetitions, italics; 5' non-coding regions, lower case letters; coding regions, capital letters; ATG start codons, boxed.

a

```
Er-msrA1  ccccaaaaccccaaaaccccaaaaccccgtacgtgtaaactgactcaatt-cttgctaaaatttataat-at---taaaa----------
Er-msrA2  ccccaaaaccccaaaaccccaaaacccctatttaggagggatggctgaggagtataataaattattaaatATGGTTAAAAGAGGCGG-AT
Er-msrA3  cccccaaaaccccaaaaccccaaaacccc----------------tcaattac--tttcaaacg--ttga-ATGCATAAAAGAATGAGTAT

Er-msrA1  -----------------------------------------------------------------------------------tatggag
Er-msrA2  TTAGAGGCAGACGTAGTAGCCGTGGCAGATCTAGAGGTGGAAGAGGCGGCAGAGGTAGAAGAGATGATGAAGGCTTCAAGAACAACCGCA
Er-msrA3  TCCAAACTATTCCGATTTGATTATT-----------------------------------GAGAG-ATCTTGAT-----GAAT-------

Er-msrA1  gccgaaggttacggcgaatttcttaaccaagtggaggaagtatcATGACTGAACGTGCTGT-ACTGGCAGGAGGCTGCTTCTGGGGCATG
Er-msrA2  GAGGCCGTGGAGGTTTTAGAGGTAAGCCAAGAGGAGGCAGTATCATGACTGAACGTGCTGT-ACTGGCAGGAGGCTGCTTCTGGGGCATG
Er-msrA3  -----------------------------------------------------TGTGAGGTCAGTGGCTGGAGGCTGCTTCTGGGGCATG

er-msrA1  CAGGACCTGATCCGCAAGAAGCCCGGCGTGGTTTCCACACGTGTGGGATATACCGGCGGCGATGTGCCTAACGCTACTTACCGCAACCAC
er-msrA2  CAGGACCTGATCCGCAAGAAGCCCGGCGTGGTTTCCACACGTGTGGGATATACCGGCGGCGATGTGCCTAACGCTACTTACCGCAACCAC
er-msrA3  CAGGACCTGATCCGCAAGAAGCCCGGCGTGGTTTCCACACGTGTGGGATATACCGGCGGCGATGTGCCTAACGCTACTTACCGCAACCAC

er-msrA1  GGAGATCATGCCGAGGGGATCGAGATCATCTTTGACCCCGACCGCATCAGCTACCGTGATCTTCTG 330
er-msrA2  GGAGATCATGCCGAGGGGATCGAGATCATCTTTGACCCCGACCGCATCAGCTACCGTGATCTTCTG 424
er-msrA3  GGAGATCATGCCGAGGGGATCGAGATCATCTTTGACCCCGACCGCATCAGCTACCGTGATCTTCTG 304
```

b

```
Er-MsrA1 ---------------------------------------------------MTERAVLAGGCFWGMQDLIRKKPGVVSTRVGYTGG
Er-MsrA2 MVKRGGFRGRRSSRGRSRGGRGGRGRRDDEGFKNNRRGRGGFRGKPRGGSIMTERAVLAGGCFWGMQDLIRKKPGVVSTRVGYTGG
Er-MsrA3 MHKR------MSIPNYS-------------DLIIER-------------SCCIVRSV-AGGCFWGMQDLIRKKPGVVSTRVGYTGG

Er-MsrA1 DVPNATYRNHGDHAEGIEIIFDPDRISYRDLL 67
Er-MsrA2 DVPNATYRNHGDHAEGIEIIFDPDRISYRDLL 118
Er-MsrA3 DVPNATYRNHGDHAEGIEIIFDPDRISYRDLL 85
```
