# Supplementary material for: Evidence for methionine-sulfoxide-reductase gene transfer from Alphaproteobacteria to the transcriptionally active (macro)nucleus of the ciliate, Euplotes raikovi
Source: BMC Microbiol. 2014 Nov 25;14:288. doi: 10.1186/s12866-014-0288-1 (PMC4247871; doi:10.1186/s12866-014-0288-1)

Additional file 3

Figure S2. (a) Schematic representation of the *msrAB* gene showing the relative positions of the three ORF's and PCR primers (sequences in the Additional file 1: Table S1) used for assessing ORF expression. Non coding 5' and 3' regions and telomeric repetitions at the gene extremities are represented by grey and black boxes, respectively. (**b**) Separation on agarose gel of PCR products (arrows) obtained by cDNA amplifications run with the indicated primer combinations.

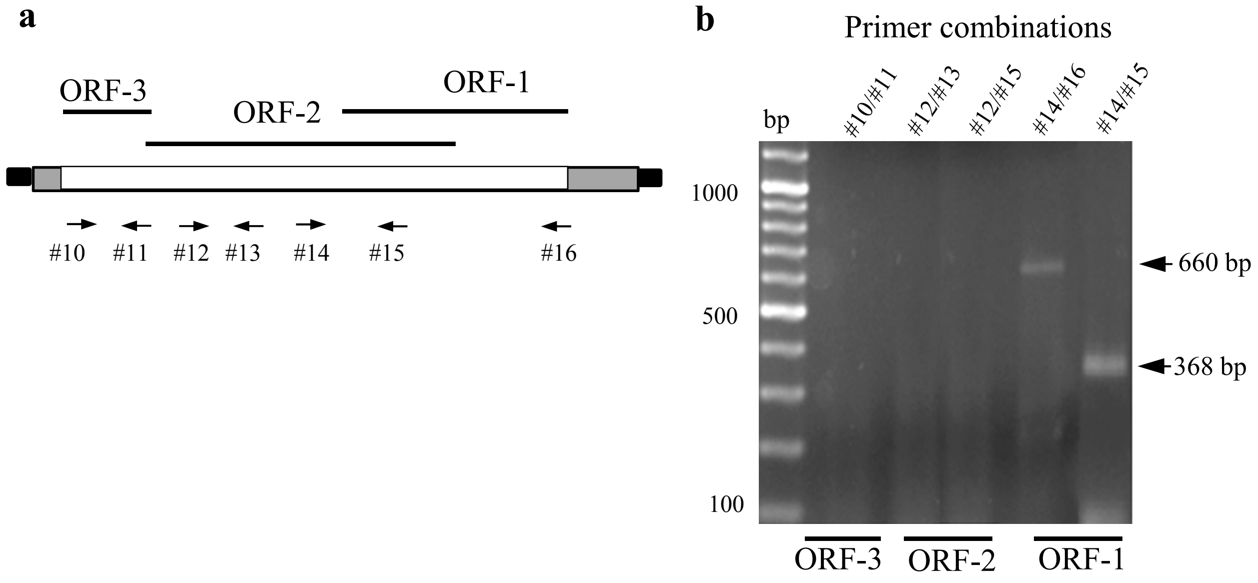

Supplement: Additional file 3: Figure S2. — (a) Schematic representation of the msrAB gene showing the relative positions of the three ORF’s and PCR primers used for assessing the expression of each ORF. (b) Agarose-gel separation of PCR products obtained from cDNA amplifications run with the indicated primer combinations. [file 12866_2014_288_MOESM3_ESM.pdf]
