# Supplementary material for: Evidence for methionine-sulfoxide-reductase gene transfer from Alphaproteobacteria to the transcriptionally active (macro)nucleus of the ciliate, Euplotes raikovi
Source: BMC Microbiol. 2014 Nov 25;14:288. doi: 10.1186/s12866-014-0288-1 (PMC4247871; doi:10.1186/s12866-014-0288-1)

Additional file 4

Figure S3 Nucleotide sequence alignment between *E. raikovi msrAB* gene (from nucleotide 305 to nucleotide 1,268) and *Thalassobacter arenae* chromosome (from nucleotide 543,304 to nucleotide 544,253). Gaps were inserted to maximize the alignment. Start and stop codons are highlighted in gray. The in-frame TGA codon is boxed. MsrB and MsrA coding regions are indicated by green and red bars above the *E. raikovi msrAB* sequence, and below the *T. arenae* chromosome sequence. Asterisks indicate identical nucleotides.

```
msrAB       ATGACATATAAAAAATCGGACGACGCTATTGCCAATCTGACACCGGAGCAATTCCGCGTGACTCAGGAAAATGGAACTGAGCGCCCATAT
T. arenae   ATGAAATACGCCAAGACCGATGAAGCCCTTGCCAAGCTCACCCCCGAACAGTTCCGCGTGACGCAGGAAAACGGCACCGAACGCGCCTTC
            **** ***    **  * ** ** **  ******* ** ** ** ** ** *********** ******** ** ** ** *** * *

msrAB       ACTGGCGAGTACTACAAAAACAAAGAACCCGGCATTTATGTTGATATCGTCTCCGGCGAACCGCTCTTCGCTTCGCCGACAAGTTTGACG
T. arenae   ACCGGGGAGCTGACCGACAACAAGGAGCCCGGCATCTACGTCGACATCGTGTCTGGCGAGCCGCTGTTCGCGTCGTCCGACAAGTTCGAA
             ** ** ***    * * ***** ** ******** ** ** ** ***** ** ***** ***** ***** *** **********  **

msrAB       AGTTTTTGCGGATGGCCGAGCTTTACAAAACCGATTGTGTCGGAGCATGTCAATGAACTGCGCGATACCAGCCACGGCATGATACGGACC
T. arenae   TCCGGCTGCGGCTGGCCCAGCTTCACCAAGCCCATCGTCACAGATCACGTGACCGAACACCGCGACGCATCCTTCGGGATGGTCCGCACA
                  ***** ***** ***** ** ** ** ** **  * ** ** ** *  ****  *****  *   *  *** *** * ** **

msrAB       GAAGTGCGTTCAACCTATGGCGACAGCCATTTGGGACATGTTTTTAATGACGGCCCAAGCGAG-GCAGGTGGTCTGCGCTATTGTATCAA
T. arenae   GAGGTGCGGTCCATGCATGGGGACAGCCATCTAGGCCACGTCTTCCCTGACGGCCCCGCCGATCGCGGG-GGCCTGCGCTATTGCATCAA
            ** ***** ** *   **** ********* * ** ** ** **   *********   ***  ** ** ** *********** *****

msrAB       CTCTGCCAGCCTACGGTTTATCCATAGAGACGATATGGAGGCCGAAGGTTACGGCGAATTTCTTAACCAAGTGGAGGAAGTATCATGACT
T. arenae   CTCCGCCAGCCTGCGCTTCATTCACCGCGACGAGATGGAGCAGGAAGGCTACGGCGAATATCTGGATCAAGTGGAGGAAATCGAATGACT
            *** ******** ** ** ** **  * ***** ******   ***** ********** ***  * ************ *   ******

msrAB       GAACGTGCTGTACTGGCAGGAGGCTGCTTCTGGGGCATGCAGGACCTGATCCGCAAGAAGCCCGGCGTGGTTTCCACACGTGTGGGATAT
T. arenae   GAACGCGCTGTTCTGGCCGGTGGCTGCTTCTGGGGCATGCAGGACCTGATCCGCAAAATGCCCGGCGTTGTGTCCACCCGCGTGGGCTAC
            ***** ***** ***** ** *********************************** * ********* ** ***** ** ***** **

msrAB       ACCGGCGGCGATGTGCCTAACGCTACTTACCGCAACCACGGAGATCATGCCGAGGGGATCGAGATCATCTTTGACCCCGACCGCATCAGC
T. arenae   ACCGGCGGCGATGTGGAAAACGCCACCTACCGCAACCACGGCACCCATGCGGAAGGGATCGAGATCATCTTCGACCCTGACGTGATGAGC
            ***************   ***** ** **************    ***** ** ***************** ***** ***   ** ***

msrAB       TACCGTGATCTTCTGGAGTTTTTCTTCCAGATCCACGATCCGGCAACGCGGGACCGGCAGGGCAACGACCTCGGC-CGCAGCTATCGTTC
T. arenae   TATCGCAAGCTGCTGGAATTCTTCTTCCAGATCCATGATCCGACGACGCCGAACCGGCAGGGTAATGACCGTGGCATGTCG-TATCGGTC
            ** **  * ** ***** ** ************** ****** * **** * ********** ** ****  ***  *  * ***** **

msrAB       GGCGATATATTATGTGGACGACGCGCAAAAAGAGGTCGCGCTCGACACCATCGCCGATGTGGATGCTTCGGGTCTTTGGCCGGGTAAGGT
T. arenae   TGCCATCTACTACGTCGACGAGACGCAGAAAGAGGTGGCATTGGACACCATCGCCGACGTAGATGCCTCCGGCCTGTGGCCGGGCAAGGT
             ** ** ** ** ** *****  **** ******** **  * ************** ** ***** ** ** ** ******** *****

msrAB       TGTGACTGAAGTGGAGCCTGTAGGCGACTTCTGGGAAGCAGAACCCGAGCACCAGGATTACCTGGAGCGGTTGCCTAACGGCTATACCTG
T. arenae   GGTGACCGAGGTGGAACCCGTAGGCGATTTCTGGGAGGCCGAGCCGGAGCATCAGGACTATCTGGAACGCATCCCCAACGGCTACACCTG
             ***** ** ***** ** ******** ******** ** ** ** ***** ***** ** ***** *   * ** ******** *****

msrAB       CCATTTCCCGCGTCCCGACTGGGTACTGCCGAAACGGTCGGCGGCGGAGTAA
T. arenae   CCATTTCGTGCGCCCAGACTGGGTGCTGCCCAAACGCGCAGCGGCTGAATAG
            *******  *** ** ******** ***** *****  * ***** ** **
```

Figure S4 Nucleotide sequence alignment between *E. raikovi msrAB* gene (telomeric C_4A_4-G_4T_4 repeats excluded) and *Sinorhizobium meliloti* plasmid pRmeGR4c (from nucleotide 344,364 to nucleotide 345,900, complement sequence). Gaps were inserted to maximize the alignment. Start and stop codons are highlighted in gray and in-frame TGA are boxed. The three ORFs are indicated by blue, green and red bars above the *E. raikovi msrAB* sequence, and below the *S. meliloti* plasmid pRmeGR4c sequence. Asterisks indicate identical nucleotides.

```
msrAB         TATTGATCAGAAAAACATTGAATATTATTATAATT-TGCATGGCCTTACGATGCGTCCGGTGGAGGATATGAACTTGTCGCGTGAGGTCG
S. meliloti   GTGCGATCTGCATCATGCCCGAGCGGTCAATCGTCGTGCAGGGCATCGTTACCCGCCCGGTGGAAGGGATTTCCCTGGCGCGCGAACTCG
                  **** * *  *      *        **  *  **** *** *    *  **  ******** *  **   * ** **** **  ***

msrAB         TACTCGTCACGGTATCAGGTTCGGGAATACCTATCGAAATTCGTCAGATTGTACGCATGGCTAAAGAGTATGATTGGTGGCCGGAACGAA
S. meliloti   TATTCGTGACGGTCTCCGGTTCCGGCACGCCACTCGAGATCCGCAAGATCGCGCAGCTGGCGGCGCGGTACGGCTGGCCATGAACGCGAT
              ** **** ***** ** ***** ** *  **   **** ** **  **** *  *   ****        *** *  ***         ***

msrAB         CCTCAATAGGTTGCCGGTGCTATTGAATCTATGTCCATAGGCTATTGGTAATCATAGCGGTTTTAATCCATATTCAGTTCAACGGCGAAA
S. meliloti   AGGTGCTCCAGTCGCGCGGCTATCGAATCTATGCACACCCATTATTGGGAAAACTCATCGCCGCTCGCCATATTCAGG-CAACAATGCAG
                    *    *  **  ***** *********  **      ****** **   *     *       **********  ****   * *

msrAB         ATGCCTAATGGAGAATAAAGCCATGACATATAAAAAATCGGACGACGCTATTGCCAATCTGACACCGGAGCAATTCCGCGTGACTCAGGA
S. meliloti   G-----AACGGAGATAAGGGCAATGACCTACAGAAAGACCGACGATGCGGTCCGGAAGCTGACGCCTGAGCAGTATCGGGTGACCCAGCA
                    ** *****  *  ** ***** ** * ***  * ***** **  *    ** ***** ** ***** *  ** ***** *** *

msrAB         AAATGGAACTGAGCGCCCATATACTGGCGAGTACTACAAAAACAAAGAACCCGGCATTTATGTTGATATCGTCTCCGGCGAACCGCTCTT
S. meliloti   GAACGGCACTGAACGCCCCTTCACCGGGGAGTATAATGACAACAAGGAGCCGGGAGTCTATGTCGACATCGTATCCGGCGAACCGCTGTT
               ** ** ***** ***** *  ** ** *****   *  * ***** ** ** **  * ***** ** ***** ************** **

msrAB         CGCTTCGGCCGACAAGTTTGACAGTTTTTGCGGATGGCCGAGCTTTACAAAACCGATTGTGTCGGAGCATGTCAATGAACTGCGCGATAC
S. meliloti   CGCCTCGGCTGACAAGTTCGATTCCGGCTGTGGCTGGCCAAGCTTTACCAAGCCGATCGTGTCGGCAAACGTCAACGAGTTGCGCGACGA
              *** ***** ******** **        ** ** ***** ******** ** ***** *******   * ***** **  *******

msrAB         CAGCCACGGCATGATACGGACCGAAGTGCGTTCAACCTATGGCGACAGCCATTTGGGACATGTTTTTAATGACGGCCCAAGCGAGGCAGG
S. meliloti   CTCCTATGGCATGACCCGCACCGAGGTGCGCTCGGTGTTTGGTGACAGCCACCTTGGTCATGTGTTTCCCGATGGTCCAGAAGACCGGGG
              *  * * *******  ** ***** ***** **     * *** ********  * ** ***** ***    ** ** ***    **     **

msrAB         TGGTCTGCGCTATTGTATCAACTCTGCCAGCCTACGGTTTATCCATAGAGACGATATGGAGGCCGAAGGTTACGGCGAATTTCTTAACCA
S. meliloti   TGGGTTGCGGTACTGCATCAATTCCGCCGCACTCCGGTTCATTCCCCGCGAGGAAATGGAGGCCGAGGGCTACGGCGCGTTCACCAACCA
              ***   **** ** ** ***** ** ***    ** ***** ** *    * ** ** *********** ** *******  **     *****

msrAB         AGTGGAGGA----------AGTATCATGACTGAACGTGCTGTACTGGCAGGAGGCTGCTTCTGGGGCATGCAGGACCTGATCCGCAAGAA
S. meliloti   GGTGGAGGATATCTGAATGAGTGAGAAACCTGAGAGAGCTGTTTTGGCTGGTGGGTGCTTCTGGGGGATGCAGGACCTGATCCGCAAGCT
               ********          ***    *    ****  * *****    **** ** ** *********** *********************

msrAB         GCCCGGCGTGGTTTCCACACGTGTGGGATATACCGGCGGCGATGTGCCTAACGCTACTTACCGCAACCACGGAGATCATGCCGAGGGGAT
S. meliloti   CCCCGGCGTCATCGAGACCCGCGTGGGCTACACTGGCGGCGACGTGCCGAATGCGACCTACCGCAATCACGGCACGCACGCCGAAGGCAT
               ********  *    ** ** ***** ** ** ******** ***** ** ** ** ******** *****    ** ***** ** **

msrAB         CGAGATCATCTTTGACCCCGACCGCATCAGCTACCGTGATCTTCTGGAGTTTTTCTTCCAGATCCACGATCCGGCAACGCGGGACCGGCA
S. meliloti   CGAGATCATCTTCGATCCCGAGAGGATCAGCTACAGGCAGATCCTGGAGCTGTTCTTCCAGATCCACGATCCGACTACGAAGAACCGACA
              ************ ** *****  * ********* *   *  * ****** * ********************* * ***   * **** **

msrAB         GGGCAACGACCTCGGCCCGCAGCTATCGTTCGGCGATATATTATGTGGACGACGCGCAAAAAGAGGTCGCGCTCGACACCATCGCCGATGT
S. meliloti   GGGCAACGACATCGGGACCTCCTACCGCTCGGCGATCTACTATGTCGACGACGAGCAGAAGCGAATTGCCCAGGAGACGATTGCAGACGT
              ********** ****  *   *** ** ******** ** ***** ******* *** **      * ** *  ** ** ** ** ** **

msrAB         GGATGCTTCGGGTCTTTGGCCGGGTAAGGTTGTGACTGAAGTGGAGCCTGTAGGCGACTTCTGGGAAGCAGAACCCGAGCACCAGGATTA
S. meliloti   CGAGGCCTCTGGCCTATGGCCGGGCAAGGTCGTGACTGAAGTCGAGCCGGTGGGAGACTTCTGGGAGGCGGAGCCGGAGCACCAGGATTA
               ** ** ** ** ** ******** ***** *********** ***** ** ** *********** ** ** ** **************

msrAB         CCTGGAGCGGTTGCCTAACGGCTATACCTGCCATTTCCCGCGTCCCGACTGGGTACTGCCGAAACGGTCGGCGGCGGAGTAAAGATCAG
S. meliloti   CCTGGAGCGCTATCCCAACGGCTACACCTGCCACTTCCCGCGCCCCAACTGGGTGCTACCCCGGCGGTCCGCGGCCGAGTAAACGGCCGA
              ********* *  ** ******** ******** ******** *** ******* ** **     ***** ***** ******* *  *

msrAB         ACACACAGCAGGGCGGTAAGGCCGCCCTGCATTTCCAATGCCATCCGAGAGGAGAGGCTAGAAAGGAGCATGTCATGCAATTTGGAGGGC
S. meliloti   AGATTTTCGCCACC--TAAAGCAACTCCGA-----CGGGCCTTGCGGATACGCCGGGCTCCTCGGAAAACACTCGGGCAGTTTCCCTCGC
              * *          *  *** **  * * *       *     *    * ** * *   ****      * *      **  *** ***      **

msrAB         TTTACGAGCAGGCAAAAGAACTACGTGTGGCGCAGTGGATCGACGGCGGCGGCAAGCCGA-TGGATGCT--CCTTTG-AGAGCTTTCCCT
S. meliloti   TACCGGCTGCGCTCCGCGAGCTACGC-TGAC-CAGGGATTCGAACGATGACGACTTCCGCCTAGGCGGTAGCCGTTGCAGATGTAAAATA
              *    *     *       ** *****  ** * *** *  ****   *   *   *      ***  * *  * *  ** *** ***  *

msrAB         GAGCGATCTGGGAAGTGGCTACAAGGTGATCTATTGCTTCCAGCACTGGTGTCCGGGGTGTAATTCGCGCGGCTTTCCAACGCTGAAATA
S. meliloti   CTTATCTGCAGCGGAGGG-TCGTTACCATGGCATTCAATACGCAACGGCT--CCAATTTTCCGGGCA-------TTCCGGCGCGACGCTT
                    *   *     ** *              ***   * *   ** * *  **     *       *         ****  ***      *

msrAB         CTTGTATCAGCGATTGAACGA-TCGCGGTGTTGG
S. meliloti   AGC-GCCCGCCTCGATCTGCCAAATGGGCCATTG
                     *  *              **   * *
```

Figure S5 Nucleotide sequence alignment between *E. raikovi msrAB* gene (from nucleotide 305 to nucleotide 1,280) and *Sphingopyxix alaskensis* chromosome (from nucleotide 121,993 to nucleotide 122,954). Gaps were inserted to maximize the alignment. Start and stop codons are highlighted in gray. The in-frame TGA codon is boxed. MsrB and MsrA coding regions are indicated by green and red bars above the *E. raikovi msrAB* sequence, and below the *S. alaskensis* chromosome sequence. Asterisks indicate identical nucleotides.

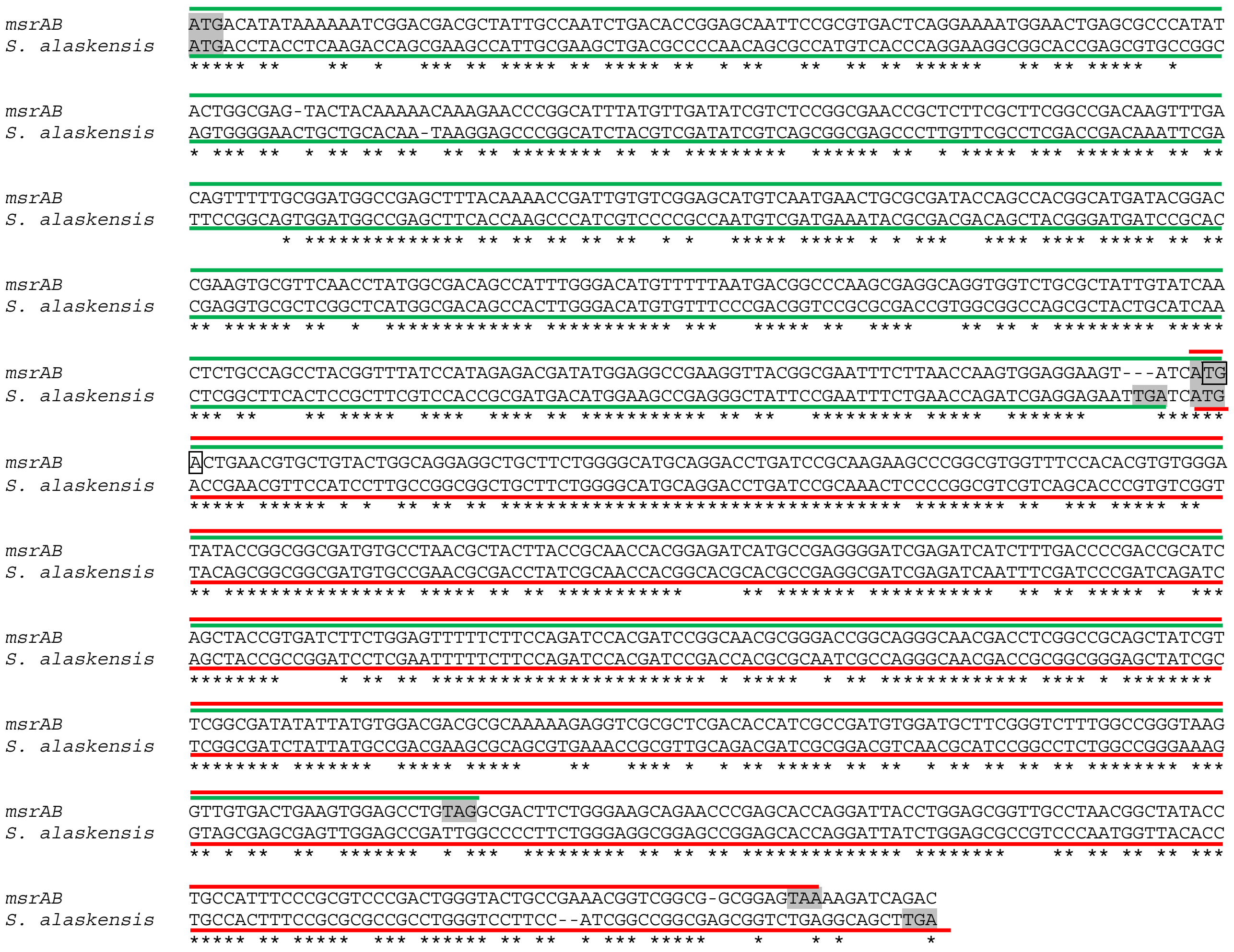

Supplement: Additional file 4: Figure S3. — Nucleotide sequence alignment between E. raikovi msrAB gene (from nucleotide 305 to nucleotide 1,268) and Thalassobacter arenae chromosome (from nucleotide 543,304 to nucleotide 544,253). Figure S4. Nucleotide sequence alignment between E. raikovi msrAB gene (telomeric C4A4-G4T4 repeats excluded) and Sinorhizobium meliloti plasmid pRmeGR4c (from nucleotide 344,364 to nucleotide 345,900, complement sequence). Figure S5. Nucleotide sequence alignment between E. raikovi msrAB gene (from nucleotide 305 to nucleotide 1,280) and Sphingopyxix alaskensis chromosome (from nucleotide 121,993 to nucleotide 122,954). [file 12866_2014_288_MOESM4_ESM.pdf]
